# Physical Examination and Appendiceal Signs During Pregnancy

**DOI:** 10.7759/cureus.22164

**Published:** 2022-02-13

**Authors:** Steven Yale, Halil Tekiner, Eileen S Yale

**Affiliations:** 1 Internal Medicine, University of Central Florida College of Medicine, Orlando, USA; 2 Medical History and Ethics, Erciyes University Faculty of Medicine, Kayseri, TUR; 3 General Internal Medicine, University of Florida College of Medicine, Gainesville, USA

**Keywords:** physical examination, rovsing sign, cope sign, bryan sign, alders sign

## Abstract

The clinical diagnosis of acute appendicitis is challenging as patients present with an array of objective and subjective symptoms early or late in the disease course. Ultrasound is routinely performed in all patients with suspected acute appendicitis. Equivocal test results frequently require further assessments using other imaging techniques that are limited in scope during pregnancy because of issues involving safety, availability, and accessibility. Physical examination diagnostic signs in acute appendicitis during pregnancy have not been well studied. Studies failed to describe, standardize, or correlate the technique used to the pathologic disease process. Therefore, gaps remain in current knowledge regarding the usefulness and application of these tests during the physical examination. Improvement in diagnostic acumen is critically important, particularly in cases where there remains diagnostic uncertainty because of equivocal imaging results. This article reviews signs used to diagnose patients with acute appendicitis using a pathophysiologic approach based on visceral and cerebrospinal nerve pathways to explain the mechanism for a positive test result. It also suggests a framework to study them further to better understand their role, if any, in clinical practice.

## Introduction and background

Acute appendicitis during pregnancy is a common cause of nonobstetrical abdominal pain. In some cases, especially during the early stages of the disease, the diagnosis remains elusive despite advanced imaging techniques. Thus, a comprehensive history and physical examination with serial evaluations remain the cornerstone in assessment and diagnosis. Gaps in knowledge remain regarding the role of the physical examination in diagnosis. The purpose of this article is to describe the physical examination signs reported during pregnancy, the pathophysiologic mechanism involved in a positive test result, and a framework for future studies assessing their effectiveness.

Various tools have been studied to confirm a diagnosis of acute appendicitis during pregnancy. A recent retrospective case series in 42 pregnancies with acute appendicitis found that laboratory studies alone did not distinguish acute appendicitis from nonappendiceal disease [1]. Clinical predictive models such as the Alvarado and Tzanakis clinical scores are limited as they were not developed for acute appendicitis during pregnancy and contain points for laboratory data (e.g., leukocytosis) which some studies found were not useful distinguishing feature during pregnancy [1-2]. Ultrasonographic imaging has been reported to have moderately high sensitivity and specificity and is the initial method of choice in diagnosis [3]. In cases where the results are inconclusive and clinical suspicion remains, MRI is preferred and recommended as it avoids ionizing radiation to the fetus by CT scanning and generally is a test with high sensitivity and specificity [4-6]. In cases of low or intermediate probability of disease, patients can either follow-up with ultrasound in six to 12 hours or proceed to MRI. Unfortunately, MRI is limited in that it imposes challenges related to accessibility and availability. Thus, the diagnosis of acute appendicitis, particularly during pregnancy, may remain elusive despite laboratory and imaging studies [3]. Bedside modalities that enhance the pretest probability of disease would better direct the urgency of the evaluation.

## Review

Cerebrospinal and visceral nerve pathways

Different descriptions and locations of the pain in acute appendicitis can be explained by whether the splanchnic (visceral) and/or cerebrospinal (somatic) nerve pathways are involved [7-8]. In acute appendicitis early in the disease course, Pacinian mechanoreceptors and splanchnic afferent nerve fibers within the appendix are activated in response to stretching, spasms, or compression [7]. The pain fibers travel with splanchnic sympathetic nerves (superior mesenteric, gastric, and hepatic plexus) to the dorsal root ganglion. The visceral efferent pain fibers, in general, travel along the same spinal cord segments as their afferent nerve counterparts. Visceral pain is perceived in the midline (epigastric and periumbilical regions), at the T8-T10 dermatomes, reflecting its embryologic nerve origin. This viscerosensory reflex is generally more widespread, poorly localized, bilaterally distributed, and extends to involve several body segments. Hence, the location of this pain in early appendicitis is fixed regardless of the position of the appendix. What is not frequently recognized is that in some cases, when the pain is abrupt and severe, there is “spill-over” of splanchnic to somatic afferent neurons given their close anatomical proximity in the dorsal root of the spinal cord [7-8]. Because of this phenomenon, severe visceral appendiceal pain may also directly activate alpha motor neurons and efferent nerve pathways in intercostal nerves that innervate the abdominal wall muscles producing involuntary abdominal spasm (guarding) and pain at the right T10-T11 dermatome in the absence of inflammatory involvement of the parietal peritoneum. This latter phenomenon represents visceromotor and viscerosensory segmental reflexes.

In contrast, inflammation of the parietal peritoneum of the abdominal wall activates cerebrospinal nerve pathways with pain afferent signals transmitted through intercostal nerves to the dorsal root ganglion (somatosensory segmental reflex). Additionally, movement, cough, removal of the palpatory hand, or other maneuvers activate anterior spinal root alpha neurons that innervate the abdominal wall muscles causing spasm and guarding (somatomotor segmental reflex). This reflex localizes the pain to the same area at the inflamed peritoneum. In patients with acute appendicitis, with disease involving the parietal peritoneum, pain most commonly occurs at McBurney point, the site “very exactly between an inch and a half and two inches from the right anterior spinous process of the ilium on a straight line drawn from that process to the umbilicus" (p. 678) [9]. It is essential to recognize that first, this site does not represent the position of the appendix but rather the location on the anterior abdominal wall where the nerve endings of T11-T12 dorsal segments are most commonly reflexively irritated by appendiceal inflammation [10]. Second, other points of maximal tenderness on the anterior abdominal wall have been reported by Cope, Lanz, Clado, and Gray [10]. Third, pain at McBurney point is not present when the appendix is located retrocecal, in the pelvis, or involving the posterior abdominal wall. In these cases, the pain fibers are the lumbosacral rather than thoracic cerebrospinal nerves [10]. Fourth, somatic pain is less likely present at this site during the third trimester of pregnancy if the appendix is displaced or the space between the appendix and abdominal wall is increased [8,10].

Signs described in acute appendicitis

Physical examination findings in acute appendicitis, if reported, mainly assessed symptoms of abdominal wall pain and rebound tenderness elicited during abdominal palpation [11-17]. Some studies further included Rovsing, psoas, and/or obturator signs and concluded that they lacked sufficient utility in diagnosis [12,14-15]. It is important to recognize that these signs were not originally explicitly used to diagnose acute appendicitis during pregnancy. Studies neither described how the signs were performed nor standardized the technique used among examiners. Furthermore, the pathologic stage was not correlated with physical findings. In other words, it is unknown whether the disease process was confined to the appendix or progressed to involve the parietal peritoneum secondarily. This is important particularly to know, for example, in the case of the psoas sign where a positive test requires the inflammatory process to extend and involve the psoas muscle [18].

Standardization of technique is essential to ensure reproducibility and consistency in reporting when evaluating the sensitivity or specificity of a bedside sign. To assess the extent of this problem, Prosenz and Hirtler evaluated the accuracy of the description of Rovsing sign in English and German literature [19]. None of the English studies or textbooks evaluated in this study included a correct description of the sign [19]. The sign, as described by Rovsing in 1907, is as follows: I wondered whether I could elicit the typical pain in the right iliac fossa by applying pressure at the left iliac fossa. This involves compressing the descending colon by pushing the fingers of my right hand onto the fingers of the left hand placed flat against the abdomen in the left iliac fossa. Using this method, the hands slide upward toward the left colonic flexure (p. 1258) [20].

Thus, Rovsing sign involves activation of Pacinian receptors in the inflamed appendix in response to distension by the retrograde flow of gas in the colon. In this case, afferent nerve fibers carried through splanchnic sympathetic nerves ultimately activate visceral efferent pathways resulting in pain in the right lower quadrant at the T10-T11 dermatome (viscerosensory segmental reflex). Variations in the technique when performing this sign incorrectly described it as using one or both hands applying only downward pressure without a sliding motion in the left lower quadrant or the same maneuver followed by a quick release of the hand from the abdominal wall (rebound tenderness) [21-24]. The latter finding is referred to as the Blumberg sign, a maneuver used to detect inflammation of the parietal peritoneum [25].

The Psoas sign, as described by Cope in 1921 [18], is a method for “determination of iliopsoas rigidity” and is performed as follows: It is well known that if there is an inflamed focus in relation to the psoas muscle, the corresponding thigh is often flexed by the patient to relieve the pain. A lesser degree of such contraction (and irritation) can be determined often by making the patient lie on the opposite side and extending the thigh on the affected side to the full extent. Pain will be caused by the maneuver if the psoas is rigid from either reflex or direct irritation (p. 42) [18].

Variations of this test that are erroneously used in clinical practice include having the patient positioned supine followed by flexion of the thigh against the examiner’s hand placed above the knee [23]. The test is most likely positive in cases of retrocecal appendicitis or primary or secondary psoas abscess arising from the appendix. Conversely, it is more likely negative in cases where the disease is subacute, or the abdominal wall is rigid [26]. Knowledge of factors that lead to a false positive and false negative test accounts for the variability in reporting on the sensitivity and specificity of this sign.

Finally, the thigh rotation or obturator test is another test described by Cope in cases of acute appendicitis: The right thigh is slightly flexed (so as to relax the psoas muscle) by the surgeon, who stands on the right of the patient; the limb is then fully rotated at the hip, first internally and then externally, so as to put the obturator internus through a full range of movement. The sign is positive if the patient complains of hypogastric pain when the limb is moved in this manner (p. 537) [27].

A positive test requires that the inflamed appendix be located below the pelvic brim, adherent to or contiguous with the fascia of the obturator internus muscle [27]. Several unique features must be borne in mind when using the psoas and obturator tests. A positive test result indicates that the disease process is located either retroperitoneally or in the pelvis; nerve innervation of structures is by lumbosacral nerves, not the intercostal nerve found in the abdomen; and pain is not distributed along a spinal segment on the anterior abdominal wall. The location of the pain caused by the inflammatory irritation involving the psoas muscle was not delineated by Cope [18,26]. This muscle is located retroperitoneally both within the abdomen and pelvis, arises from the vertebral bodies of T12 and L1-L3, attaches to the less trochanter of the femur, and is innervated by L1-L4 nerves. Thus, depending on the location of the inflammatory process along the psoas muscle, pain may be perceived in the lower back, right abdominal quadrant, inguinal and hypogastric regions, hip, thigh, or knee but not on the right anterior abdominal wall. Conversely, involvement of the obturator internus muscle and a positive obturator sign indicate inflammation of the pelvis, where the pain is always perceived in the hypogastrium (suprapubically). These findings further emphasize that clinicians employing these maneuvers must be aware of how they should be appropriately performed, their limitations, pain location, and the significance of a positive test result.

Controversy exists regarding the anatomical location of the appendix during pregnancy. Interestingly, studies evaluating the position of the appendix during appendectomy or cesarean section found no changes in the normal location of the appendix, while imaging with CT or MRI identified upward displacement with advancing stages of pregnancy [28-31]. The physical examination may be further limited by the distance between the inflamed appendix and the abdominal wall, which widens during pregnancy and potentially reduces the probability of developing abdominal wall tenderness [13,17].

Signs for acute appendicitis specifically designed during pregnancy

Alders and Bryan described two bedside maneuvers named as signs for their namesake as a method for distinguishing uterine from extrauterine (e.g., acute appendicitis) causes specifically during pregnancy [32-33]. Alders sign is performed as follows: With the patient lying straight on her back, the examining fingers find the area of maximum tenderness to pressure on the abdominal wall. While the fingers remain in contact with that area without altering the intensity of pressure, they are exerting to elicit pain, the patient is made to turn over on the opposite side so that the plane of the anterior abdominal wall is approximately vertical. The pain produced by the pressure of the fingers will be less or will have entirely disappeared if the lesion is uterine and has fallen away from the examining fingers, “shifting tenderness” (pp. 1194-1195) [32].

Alders sign is based on the anatomic principle that the appendix is a retroperitoneal structure, remains fixed, and does not shift when the patient moves to the left decubitus position [32]. In contrast, pain arising from the uterus resolves as the patient moves from a supine to the left decubitus position, away from pressure exerted by the examiner’s hand. Although not explicitly stated by Adler [32], but based on the principles previously enumerated, this maneuver elicits a viscerosensory segmental reflex that persists regardless of the patient’s position when “deep” pressure from the examining fingers is applied in cases when the appendix is inflamed.

Bryan also reported a method for diagnosing appendicitis during pregnancy and the puerperium such that applying “pressure on the pregnant uterus from the left side will often elicit pain in the right lower or middle quadrants” (p. 1205) [33]. The presence of pain at the right lower or right middle abdominal quadrants suggests activation of visceral afferent nerve fibers and a viscerosensory segmental reflex caused by the gravid uterus shifted to the right, compressing the inflamed appendix. Neither of these signs has been well studied. One report by Chen et al. in 14 patients found that Alders sign was present in five (36%) [34].

Summary of signs of acute appendicitis in children and adults and during pregnancy

Several studies identified in the literature to present assessed the outcomes of a positive or negative psoas, obturator, and/or Rovsing sign in children and adults and during pregnancy in patients diagnosed with and without appendicitis. Studies were prospective, retrospective, and cross-sectional in design and chosen if adequate information was available to construct a 2 x 2 contingency table to calculate sensitivity, specificity, and likelihood ratios (LRs) (Tables 1, 2).

**Table 1:** Psoas, obturator, and Rovsing signs in children and adults. LR: likelihood ratio.

Sign	Number of cases	Sensitivity	Specificity	LR+ (95% CI)	LR- (95% CI)
Psoas [35-37]	1187	0.16	0.88	1.49 (1.10-2.01)	0.94 (0.90-0.98)
Obturator [35, 37]	751	0.18	0.89	1.75 (1.2-2.56)	0.91 (0.8600.97)
Rovsing [36-39]	902	0.27	0.86	2.06 (1.68-2.52)	0.83 (0.79-0.88)

**Table 2:** Psoas, obturator, and Rovsing signs during pregnancy. LR: likelihood ratio.

Sign	Number of cases	Sensitivity	Specificity	LR+ (95% CI)	LR- (95% CI)
Psoas [14]	84	0.14	0.66	0.44 (0.20-1.0)	1.28 (0.97-1.68)
Obturator [14]	84	0.12	0.73	0.49 (0.20-1.21)	1.19 (0.93-1.51)
Rovsing [12, 14]	136	0.29	0.64	0.89 (0.52-1.52)	1.05 (0.82-1.34)

No studies defined the technique for performing these signs. Both the psoas and obturator signs share similar operating characteristics. Rovsing sign had the highest LR, which is interesting in light of questions regarding its consistency in performance [19]. In both children and adults and during pregnancy, these tests had low sensitivity and moderate to high specificity. The higher specificity suggests that a positive test increases the likelihood of having appendicitis, but a negative test does not exclude the disease. There was a minimal to a slight increase in the likelihood of these tests being diagnostic for acute appendicitis in children and adults. Hence, the tests are helpful, if present, in diagnosing acute appendicitis. In contrast, these maneuvers during pregnancy are poor in diagnosing acute appendicitis when the test result is positive. Thus, these tests during pregnancy were more helpful in ruling out rather than ruling in appendicitis. It is unknown whether a combination of these physical examination techniques improves the accuracy of these bedside diagnostic tests.

## Conclusions

Gaps remain in the current understanding of the physical examination signs during pregnancy. It is unknown whether these signs are useful in supporting the clinical diagnosis of acute appendicitis. These limitations involve several factors, including not performing the maneuver as originally intended, lack of standardization of the test, and a failure to correlate the stage of the disease with findings on clinical examination. Improved knowledge may help reduce diagnostic uncertainty, particularly in cases where there is intermediate suspicion for the presence of the disease. We recommend that these physical examination signs be included and rigorously studied in future research that assesses models, laboratory, or imaging aspects of acute appendicitis during pregnancy. In the interim, MRI of the abdomen remains the gold standard in diagnosing acute appendicitis during pregnancy.
